# Using RFID to Enhance Security in Off-Site Data Storage

**DOI:** 10.3390/s100908010

**Published:** 2010-08-27

**Authors:** Miguel A. Lopez-Carmona, Ivan Marsa-Maestre, Enrique de la Hoz, Juan R. Velasco

**Affiliations:** Department of Computing Engineering, University of Alcala, Alcala de Henares (Madrid), Spain; E-Mails: ivan.marsa@uah.es (I.M.M.); enrique.delahoz@uah.es (E.H.); juanramon.velasco@uah.es (J.R.V.)

**Keywords:** Off-site data storage, RFID, security controls

## Abstract

Off-site data storage is one of the most widely used strategies in enterprises of all sizes to improve business continuity. In medium-to-large size enterprises, the off-site data storage processes are usually outsourced to specialized providers. However, outsourcing the storage of critical business information assets raises serious security considerations, some of which are usually either disregarded or incorrectly addressed by service providers. This article reviews these security considerations and presents a radio frequency identification (RFID)-based, off-site, data storage management system specifically designed to address security issues. The system relies on a set of security mechanisms or controls that are arranged in security layers or tiers to balance security requirements with usability and costs. The system has been successfully implemented, deployed and put into production. In addition, an experimental comparison with classical bar-code-based systems is provided, demonstrating the system’s benefits in terms of efficiency and failure prevention.

## 1. Introduction

In recent years, information has become the greatest asset of many enterprises. In a world of threats, this should make information security one of the highest priorities in corporate environments. Although the different security functions (usually denoted as confidentiality, integrity and availability [1]) are given different weights in an organization’s strategic plans, there is a trend to regard the top enterprise data security issues as confidentiality [2], business continuity and regulatory compliance [3].

Business continuity refers to activities oriented to ensure that business-essential functions within an organization will continue uninterrupted during and after a disaster [4]. Among the different processes and techniques involved in business continuity planning, one of the most widely used by corporations of all sizes is *off-site data storage*, which involves the periodic backup of data and its storage in a separate location. In medium-to-large size enterprises, the off-site data storage process is usually outsourced to a specialized provider that maintains secure media vaults in which data may be safely stored.

There are many companies offering secure data storage services all over the world [5]. Most of them offer state-of-the-art requirements for storage, such as temperature and humidity control, fire-proofing or even electromagnetic shielding (so-called ‘Faraday cages’). However, there are other security considerations inherent in the very process of data backup and external storage that are often not properly addressed [6]. These are mostly related to physical threats, such as theft during the delivery of backup tapes, loss or physical damage of backup media during transit, or accidental or intentional misdeliveries (*i.e.*, delivery to other customers not authorized to access the data).

Electronic data vaulting [7] allows for automated archiving, recovery, and “online backup” of data to a remote location. Although the intent of the process is to mitigate most of the aforementioned physical risks, it in turn raises new security concerns derived from the transfer of sensitive data across the network and the subsequent loss of control over them once they leave the organization network perimeter. This possibility, along with availability and cost issues, makes electronic vaulting an unsuitable solution for many organizations, which must then rely on classical off-site data storage services. They must therefore cope with the aforementioned risks by employing additional mechanisms to enhance data security in the process.

In this article, we present SECUR, an RFID-based system for off-site data storage management. The system addresses the security issues of classic off-site data storage, providing:

- An RFID-based identification mechanism for data custody units and employees [8], which increases the speed and reliability in the identification process compared to bar-codes, the most widely used existing technology.
- A set of security controls throughout the entire life cycle of the custody unit, including two-way confirmation scans, the segregation of duties, continuous inventory and theft protection measures. We also arrange these security controls in a set of *security tiers*, which allows us to leverage security requirements with usability and cost constraints when deploying the system in a given scenario.
- A Web-based management service, which allows all agents involved (customers, operators and managers) to monitor the data life cycle in real time.

The rest of this paper is organized as follows: Section 2 presents a generic use case for an off-site data storage management system and outlines the key processes in the custody unit life cycle. Section 3 describes the SECUR system, detailing its architecture and the security controls involved in these processes. Section 4 presents a set of experiments devised to compare the proposed system with a classical bar-code-based solution, along with the results of these experiments. Finally, Section 5 summarizes our contributions and proposes future research.

## 2. The Off-Site Data Storage Process for Business Continuity Management

In enterprises, the information life cycle comprises several activities, such as creation, acquisition, cataloguing, storage, preservation and access [9]. From the business continuity management perspective, we focus mainly on storage, preservation, and a particular form of access we call *recovery*. For most medium-to-large sized companies, these activities are performed by means of backup policies and associated processes. For adequate protection against the most relevant threats, a backup solution must be available to transfer the data off-site (to protect it against major physical threats onsite), off-line (to allow for recovery from online attacks) and out-of-reach (to preserve backup data until it is needed). This can be achieved by means of an off-site data storage service, where a specialized service provider periodically delivers and retrieves backup media (usually magnetic tapes) and stores them in a secure vault.

### 2.1. Off-site data storage service use cases

As we have outlined above, most forms of off-site data storage services involve a delivery and retrieval process between a customer enterprise and a service provider. The objects of the delivery and retrieval, which may be individual backup media units (tapes, CDs, *etc.*) or sealed boxes of media, are generically called *custody units*. In the following sections, we briefly describe some delivery and retrieval use cases, which will serve as a reference for identifying the key processes in the service.

**Figure 1.**
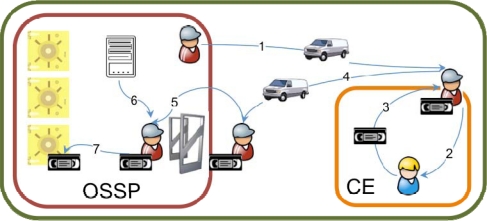
Custody unit retrieval use case.

The custody unit retrieval use case is summarized in Figure 1. An employee of an off-site storage service provider (OSSP) arrives at the customer enterprise (CE) facilities (1) either because the customer has made a specific request or because of a scheduled visit specified by contract. Once in the customer enterprise facilities, the OSSP employee meets a CE employee (2) and receives from him/her a set of custody units (3). The OSSP employee then transports the custody units (4) to the OSSP facilities, where they are identified (5), classified (6) and stored (7).

**Figure 2.**
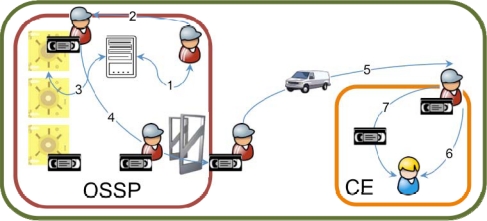
Custody unit delivery process.

The custody unit delivery use case is the opposite situation, as summarized in Figure 2. The OSSP employee obtains the location of the custody units to deliver (1), gathers them from their locations in the warehouse (2), usually registers the operation (3) and retrieves the custody units from the vault (4) to be delivered to the customer enterprise facilities (5). Once there, the OSSP employee meets with a CE employee (6) and delivers the custody unit set to him/her (7).

The description of the use cases above is a very simplified one, with no security controls in place at any step, but it serves as a starting point to try to identify the key processes as far as security is concerned.

### 2.2. Key processes and security concerns

From the use cases above, we can identify the following security processes as paramount: custody unit exchange between OSSP and CE employees, custody unit check-in and check-out to and from the vault, security within the vault, security while in transport and custody unit logical management and supervision by the involved actors. In this section, we briefly discuss the most important security considerations for each process.

*Exchange of custody units between OSSP and CE employees*. This is one of the most critical processes in both use cases because it is the point at which responsibility for the custody units is transferred between OSSP and CE. Some of the security issues that arise in this process are:

- Identification and authentication between the OSSP and CE employees because custody units must only be delivered to and retrieved from authorized personnel.
- Identification and validation of the delivered or retrieved custody units because only those custody units that have been authorized to be delivered or retrieved can be accepted. This is especially important when a single transport vehicle visits several customers because custody units must never be delivered to the wrong customers.
- Accountability of the entire process because it involves a responsibility transfer, which may include liabilities.

*Custody unit check-in and check-out at the vault.* These processes involve the transition of custody units from a highly secure environment (*i.e.*, the vault) to an environment where they will be more vulnerable to physical threats (while in transport). Therefore, only authorized personnel should be able to initiate such processes and only custody units that are intended to enter or leave the vault should do so.

*Storage within the vault and in the transport vehicle.* Most security concerns in these processes are related to physical threats and are addressed by means of physical security mechanisms, such as vault shielding or the use of unmarked transport vans. However, there are also some logistical concerns, mostly related to the segregation of duties [10].

*Supervision by the involved actors.* To maintain adequate control of these processes, OSSP authorized staff must be able to track the status of the custody units at all steps of the use case. This tracking involves confidentiality, authentication and authorization mechanisms to ensure that access to custody unit status is performed on a need-to-know basis. Finally, the ability of customers to track their own custody units may provide an additional control layer, but it may also raise new security concerns because it allows access to custody unit information from outside of the OSSP logical security perimeter.

## 3. An RFID-Based, Off-Site Storage Management System

In this section, we present SECUR, an RFID-based solution for off-site storage management that was designed to address the security considerations discussed in the previous sections. SECUR was developed with three design objectives in mind: security, modularity (to allow for granular security/usability trade-offs when needed) and interoperability. A detailed discussion of the SECUR system is beyond the scope of this article but is discussed elsewhere [11]. In this section, we will describe the general architecture of the system and discuss the different security mechanisms implemented to address the aforementioned security considerations.

### 3.1. System architecture

**Figure 3.**
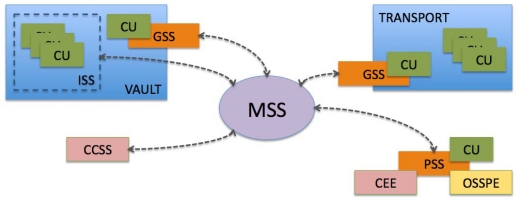
SECUR system architecture.

The SECUR off-site storage management system architecture relies on both hardware and software subsystems, which are shown in Figure 3. In this section, we briefly describe each subsystem and its interaction with the others.

*Custody Units (CU).* Each custody unit in the system (i.e., a data tape, a CD or a sealed box containing backup media) bears a passive RFID tag, which allows for its identification by other subsystems.

*OSSP Employee (OSSPE).* Each OSSP employee also has an associated passive RFID tag, which is used to identify them to other subsystems.

*CE Employee (CEE).* In a similar way, each CE employee also has an associated passive RFID tag.

*Management Subsystem (MSS).* This is the central system, which supports the operation of the rest of the processes. It maintains records of custody units, OSSP employees and CE employees, along with logs of the operations performed by the employees and any status changes. Other subsystems access the management subsystem to perform operations.

**Figure 4.**
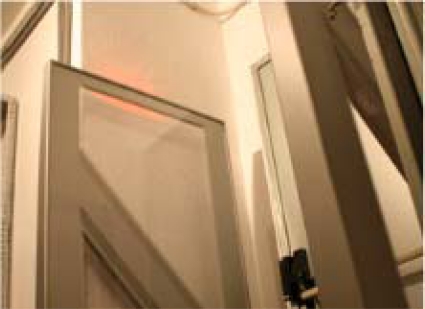
Gate Subsystem deployed in a vault.

*Gate Subsystem (GSS).* This subsystem performs physical access control for custody units and employees, allowing only authorized transitions to and from the secured vaults and transport vehicles. This subsystem may be implemented in a different way depending on where it is deployed. For instance, the vault GSS usually relies on gate RFID antennas (as seen in Figure 4), while transport RFID uses smaller antennas at the transport doors.

*Inventory Subsystem (ISS).* This subsystem may be used to provide an additional security layer for custody unit storage in the vault. It relies on a set of RFID readers to perform periodic scans of all custody units stored in the vault to detect anomalies (e.g., wrong custody unit prepared for exiting the vault) in a timely manner.

*Portable Subsystem (PSS).* This subsystem relies on a portable device (a laptop or PDA) that has connectivity to the MSS. This device is carried by OSSP employees and performs operations regarding custody units (e.g., deliveries, retrievals). The subsystem relies on portable RFID antennas to perform custody unit, OSSP employee and CE employee identifications if needed. It may also have printing capabilities (for delivery/retrieval receipts).

*Customer Client Subsystem (CCSS).* This subsystem allows customers to perform operations regarding their custody units, such as status queries or requests for deliveries. A particular concern about this subsystem is that it is outside the OSSP logical security perimeter; therefore, its interface to the MSS must be specially controlled.

### 3.2. Security mechanisms

Based on the architecture described, and given the use cases and security considerations discussed in Section II, we can devise different security mechanisms or (as named in the security audit literature) *controls*. We have grouped them into categories: communication security controls, RFID identification controls, physical access controls and process controls.

#### 3.2.1. Communication security controls

This category comprises the security mechanisms developed to ensure the confidentiality, authentication and integrity of communications between the different subsystems and, more specifically, the communications between the MSS and the remaining subsystems.

To allow interoperability with different possible implementations of the PSS and CCSS subsystems, the interface of the MSS was designed as a Web application, which ensures that (almost) any computer with a Web browser can interact with the MSS. This ease, of course, must be complemented by adequate security mechanisms. At the time of this writing, the SECUR Web interface supported the following security mechanisms:

- *TLS secure connections*, using AES-128 ciphering to provide confidentiality [12].
- *Server-side certificate* at the MSS to provide server authentication. To generate the certificate, a public-key cryptography standard (PKCS) with a 2048-bit RSA key is used [13].
- *User names and passwords* to access the MSS from the PSS and CSS to provide client authentication.
- *Subsystem client-side certificate* at the GSS and ISS to provide authentication of these subsystems for their access to the MSS. Certificate generation is analogous to the server certificate.
- *Browser client-side certificates* of the PSS and CCSS subsystems, to provide an additional layer of authentication over username/password pairs for OSSP and CE employees when accessing the MSS. Again, certificate encryption strength is the same as that used for the server certificate.

#### 3.2.2. RFID identification controls

This category includes the mechanisms intended to enable secure identification of custody units and employees by the different subsystems when necessary:

- *Plain RFID identification.* Each custody unit or employee has an associated ISO15693 RFID tag [14] with an 8 bytes serial number (UID) that can be read by the GSS and PSS subsystems to identify them to the system.
- *Password protected RFID identification.* Each ISO15693 RFID tag has an internal memory where a *protected identifier* (PID) may be stored as data. Access to this data may be protected with a 64-bit password, which provides additional security against identifier theft or replication. This prevents accidental or intentional reading of the tag by unauthorized readers. It is still vulnerable, however, to communication eavesdropping between the reader and the tag.
- *Encrypted RFID identification.* Some manufacturers provide high-security RFID tags with encryption capabilities that can store a secret key and use it to provide confidentiality and authentication in the PID transfer from the tag to the reader.

Risk assessment within the OSSP and CE organizations must be taken into account to ensure that deployed controls are adequately aligned with business goals. A higher protection will involve slower identification processes and (in the case of encryption-based solutions) increased costs of the tags and readers.

#### 3.2.3. Physical access controls

Within this category, we have included the security mechanisms put in place to control the personnel access to the secured OSSP facilities and the check-in and check-out of custody units at these facilities and at the transport vehicles:

- *Vault space division.* The vault space may be divided to provide different security levels. For instance, a vault *antechamber* may be used to temporarily store the custody units intended to be put in transit (or that recently arrived from transit), while custody units that are not being moved are kept in a more secure environment in the *inner vault*. This would work as a custody unit ‘floodgate’.
- *RFID-based vault access control*. Any of the RFID identification mechanisms described in the previous section may be used to control access to the vault. A GSS using a RFID gate antenna connected to electronically operated doors may guarantee that only authorized personnel can access each section of the vault. Combined with the vault space division described above, this can effectively provide different physical access clearance levels.
- *PIN-based vault access control.* Employees may be required to enter a *personal identification number* (PIN) to access different sections of the vault. This has the advantage that the PIN cannot be lost or stolen (like an RFID card), but it makes identification a bit more time consuming (especially if access frequency is high) and can still be vulnerable to weak PINs (e.g., ‘1234’) or oversight (also known as ‘shoulder surfing’).
- *Biometric access control.* Biometric identification mechanisms (e.g., fingerprint scans) may be used to provide access control to the vault in a more secure manner when needed.
- *RFID-based CU check-in and check-out control.* Regarding personnel access control, any of the RFID mechanisms described in the previous section may be used. This controls which custody units enter or exit the vault, allowing only authorized custody units (e.g., those that are intended to be delivered) to leave the vault with authorized personnel (e.g., the driver entitled to the delivery). In addition, similar controls can be put in transport vans to prevent mistakes when extracting custody units for delivery to a customer.
- *Continuous RFID inventory.* This involves deploying an ISS at the inner vault, which performs periodic scans on all custody units stored in the vault. In this way, positive control of custody units (*i.e.,* custody unit X has been detected in the vault) may be performed instead of negative control (*i.e.*, custody unit X has not been detected leaving the vault).

As with identification controls, a thorough risk assessment process at the OSSP and CE enterprises should play a key role in the decision about which physical access controls to deploy. While three-factor authentication (something you have, something you know and something you are) is usually regarded as the most secure authentication policy, usability may be negatively impacted. For instance, if an OSSP warehouse employee has to prepare the custody units for each transport and leave them in the vault antechamber to be retrieved by the corresponding driver and put in transit, this may involve very frequent access to and from the inner vault. Having to enter a PIN code, having his/her RFID card scanned and putting his/her finger in a reader for each access may become very tedious. The most time consuming of the authentication activities in this case is entering the PIN code, and repeated entry may make it vulnerable to shoulder surfing. However, just using an RFID card and fingerprint (two-factor authentication) provides almost the same level of security with much less impact on usability. In a similar way, RFID-based CU check-in and check-out controls in transport vans may not be critical if proper controls are implemented during custody unit interchange between the OSSP and CE employees. This must be taken into account to decide whether or not to implement such controls because implementing a GSS at each transport van may be quite expensive. More significant is the case of implementing an ISS in the inner vault. This security control provides a very high security level, allowing for the detection of even the most subtle manipulations (like removal of a custody unit RFID tag to make it leave the vault undetected), but its implementation cost would render it unsuitable for most applications.

#### 3.2.4. Process controls

This category encompasses the security mechanisms directly related to off-site storage business processes:

- *Custody unit scan at critical operations.* Custody units are the most critical assets in the off-site storage service; therefore, custody unit management mistakes must be avoided. In addition to ‘due care and diligence’ measures (such as transportation in padded cases and with adequate temperature conditions), special attention must be paid to the logical management of mistakes, such as preparing the wrong custody units for exit or delivering custody units to the wrong customer. To avoid such mistakes, any operation with custody units may be forced to involve a custody unit scan with the PSS, thus allowing the MSS to check that the operation is being performed with the right set of custody units.
- *Employee authentication at every operation.* Because the custody unit scan involves using the PSS, an additional control mechanism would be to require the employee to log in to the PSS before performing any operation. This permits the MSS to check that the operation is being performed by an authorized employee. Furthermore, to avoid session-hijacking, we can take advantage of the scan capabilities of the PSS and have the employee scan his/her RFID card, thus providing a two-factor authentication.
- *Segregation of duties.* In the security audit literature, the segregation of duties is seen as a paramount process control [15]. Basically, the segregation of duties implies that no single person has control over every stage of a critical process to prevent accidental or intentional propagation of mistakes or other irregularities in the processes. In the use cases under consideration, the segregation of duties may be implemented at different stages. For example, there may be a *warehouse employee* who prepares the custody units to be transported and leaves them in the vault antechamber, where they are retrieved by a *driver* who leaves the vault, puts the custody units in the transport and carries them to the customer facilities. Once there, a *co-driver* may be the only person entitled to take these custody units from the transport van and deliver them to the customer. An analogous segregation of duties may be implemented for the retrieval use case. If, at every step, the involved employee performs the aforementioned custody unit check, we effectively increase the security against the propagation of irregularities.
- *Retrieval/delivery receipts.* As stated in Section II, the custody unit exchange between OSSP and CE employees is one of the most critical processes in both use cases because it is the transaction where responsibility for the custody units is transferred between the OSSP and CE. For accountability and liability reasons, a receipt should be generated at each exchange that details the date and time of the exchange, the custody units exchanged and the names and signatures of the OSSP and CE employees involved in the transaction.
- *PSS-generated receipts*. Even though the use of receipts provides accountability, it may not be completely useful if it is not backed up by authentication and custody unit check mechanisms. The use of the PSS to generate the receipts may provide such mechanisms. The PSS scan capabilities may be used to authenticate the OSSP and CE employees (also to put their names in the receipt) and check that the custody units exchanged are the proper ones (and log the custody unit identifiers in the receipt). They may also provide real-time logging of the transaction via the MSS. In addition, the authentication of the CE employees through their RFID cards may be used to provide different authorization levels for special services (e.g., emergency deliveries).
- *Real-time custody unit tracking.* The PSS-based controls discussed in this section, along with the physical access controls described in the previous section, allow for every MSS operation to be logged in. This allows OSSP and CE managers to track custody units in real time, which may provide them with supervision capabilities, greatly enhancing the security of the process.

### 3.3. Security tiers

From the discussion about security mechanisms in the previous section, it can be seen that, as usually happens in security, the decision about which security mechanisms to implement is not straightforward. Since the implementation of the highest levels of security mechanisms may have a large impact on system usability and deployment costs, a risk assessment process should always be performed at the involved organizations. The results of this assessment will help to decide which controls must be put in place to adequately consider the security requirements and the business objectives of each specific scenario we may deal with. Taking this into account, we propose a set of “deployment strategies,” ranging from no security controls at all to the highest level of security. For ease of understanding, we have organized these deployment strategies into ‘Security Tiers,’ assuming that any upper-level tier includes the security mechanisms of the lower-level tiers (unless otherwise specified):

- *Security Tier 0.* No specific security controls. The processes would follow the use cases described in section II.1 without additional security mechanisms. This tier is included only as a reference and we strongly discourage its use.
- *Security Tier 1.* Includes PIN-based vault access control and retrieval/delivery receipts. It adds vault space division and segregation of duties. Custody unit identification and classification for delivery and retrieval are performed using bar codes. This is the standard security level provided by most off-site storage providers.
- *Security Tier 2.* Substitutes retrieval/delivery receipts by PSS-generated receipts using plain RFID scans. Custody unit and employee scans are only performed during the exchange between OSSP and CE employees. It adds TLS secure connections, username/password authentication, server-side certificates and subsystem client-side certificates to secure access to the MSS. It also adds real-time custody unit tracking for OSSP managers.
- *Security Tier 3.* Adds custody unit scan and employee authentication at every operation.
- *Security Tier 4.* Adds real-time custody unit tracking for CE managers and browser client-side certificates for access to the MSS from the CCSS.
- *Security Tier 5.* Substitutes PIN-based access control at the vault by RFID-based access control using plain RFID scans. It adds RFID-based check-in and check-out at the vault and involves implementing a GSS at the vault.
- *Security Tier 6.* Adds biometric access control to the vault.
- *Security Tier 7.* Substitutes plain RFID scans by password-protected RFID scans.
- *Security Tier 8.* Substitutes password-protected RFID scans with encrypted RFID scans.
- *Security Tier 9.* Adds RFID-based CU check-in and check-out at the transport vans. Involves implementing a GSS at each transport van.
- *Security Tier 10.* Adds continuous RFID inventory at the vault. Involves implementing an ISS at the vault.

Table 1 summarizes the different security tiers we have described. The greatest usability gaps occur between tiers 0–1, 2–3, and 6–7. The greatest cost gaps occur between tiers 0–1, 1–2, 4–5, 7–8, 8–9 and 9–10. We believe that for most applications, tier 5 will provide a high level of security with an acceptable impact on usability and a reasonable cost. For applications with very high security requirements, where the loss of usability and the increased cost are reasonable, upper security tiers may be implemented.

We believe that the organization of controls in Security Layers makes easier to get a general view of the trade-off between the positive and negative implications of the deployment of the different controls. It does not make the choice of controls straightforward, however. The decision about the controls to implement in a given setting is critical, and should be based on a careful risk assessment process. Since risk assessment has to consider many aspects of the specific corporate environment where the solution is expected to operate, it is not possible to provide here a comprehensive methodology for the decision about the deployment of the proposed Security Tiers. We may, however, review some of the most usual security risks regarding outsourced, off-site data storage (which were previously outlined in Section 2.2 when discussing key processes and security concerns), and map them to the different Security Tiers. Table 2 shows a summary of such correspondence between Security Tiers and mitigated risks.

Of special significance to this paper are the contributions to security directly derived from the use of RFID technology. We can see that the use of GSSs at the vault (Tiers 5+) prevents custody units from leaving the premises, even when concealed, which is a significant improvement for most scenarios. RFID tags are more difficult and expensive to forge than bar codes, and the use of RFID password protection and encryption (Tiers 7,8+) effectively ensure that custody unit and employee ID cards may not be forged. RFID access control at the transport vans (Tiers 9+) prevents custody unit exposure to unauthorized customers, even by mistake. The use of ISS (Tier 10) allows detecting anomalous custody unit manipulations within the vault, which greatly mitigates insider attacks.

Finally, we must not forget the direct *positive* impact that performance and usability have on security. Since most security mechanisms usually involve some loss in performance and usability, the contribution of the use of RFID technology to the efficiency of the different processes allows deploying additional controls without rending the processes unusable. For instance, bar-code based employee and CU identification at each operation (Tiers 3,4) is very inefficient for large amounts of CUs, which may lead to the removal of control points (e.g., vault space division) due to performance/usability reasons. The use of RFID for CU and employee scans (Tiers 5+), however, makes these processes much more efficient, allowing to introduce new control points if needed, like the access controls at the vans (Tiers 9+). Another example of the positive impact of RFID increased performance over security is the continuous inventory through the use of ISS (Tier 10), which would be unfeasible when using bar code identification.

## 4. Performance Evaluation

While most of the advantages and disadvantages of the different mechanisms proposed in the previous section in terms of security, usability and cost are fairly straightforward, there are some claims that may need the support of an empirical validation. These include the advantages in the usability and security of using RFID compared with bar code scanners for custody unit identification and classification, as well as the improvement in error detection by using PSS scans in all operations, compared to using them for the classification at the warehouse and during CSSP/CE custody unit interchange. This section is dedicated to the experimental evaluation of these issues.

### 4.1. Experimental settings

To assess the benefits of using the proposed RFID-based off-site storage management system, we compared the performance of a Tier-5 SECUR implementation, which we designed and implemented for *Business Continuity Management Ltd.* [16] against a Tier-1 basic bar code-based system. We set up a simulated environment with two transport vans, 50 customers and 100 custody units per customer. We generated a schedule of deliveries and retrievals with an average of 20 visits per day to different customers, divided between two transport vehicles (each transport vehicle was intended to visit, on average, 10 customers per day). Each visit involved the delivery of a random number of custody units (drawn from a uniform distribution within the interval [0, 50]) and the retrieval of a random number of different custody units (generated in the same way). These conditions assume a high load scenario (most off-site storage customers do not demand such a high retrieval/delivery rate), which is the scenario where performance may be most critical. Custody units were placed on shelves in such a way that both bar codes and RFID tags could be read by the corresponding reader without having to remove the custody units from the shelves. In this way, we compensated for the most serious drawback of bar codes (requiring direct sight). In the warehouse, two employees were tasked with the preparation of custody units in the antechamber, while a single driver per transport was in charge of carrying them from the antechamber to the transport van. For the CE/OSSP custody unit exchange, a single co-driver per transport was assigned. From the different processes involved, transport times and physical handling times were not considered because these times would be the same for both systems.

We also studied the effect of the proposed security mechanisms against malicious custody unit operations performed by authorized personnel. To evaluate this, we performed two different additional experiments. First, intermediary employees were put between warehouse and driver employees, and between driver and simulated customer employees. These intermediaries were entitled with the task of secretly substituting one of the custody units by an unauthorized one, to see if the legitimate warehouse and driver employees were able to detect the mistake on time. In a second experiments, intermediaries were removed, and employees were instructed to try to ‘cheat’ the system to allow unauthorized CUs out of the premises or to allow CU deliveries to the wrong customers.

### 4.2. Experimental results

Based on the experimental settings described above, we ran 100 experiments, corresponding to 100 days of the schedule generated. For each experiment, three different times were measured: *dispatch time*, which is the elapsed time during custody unit preparation and check-out (from the shelves to the transport); *exchange time*, which is the elapsed time during the visit (exchanging custody units between OSSP and CE employees) and *storage time*, which is the elapsed time of the custody unit storage at the vault (from the transport to the shelves). We also measured *error rate*, measured as the rate between incorrectly scanned custody units and the total number of custody units moved in each experiment, and *failure rate*, defined as the rate between the number of misplaced custody units (e.g., put in the wrong transport, delivered to the wrong customer) and the total number of custody units moved in each experiment. Measurements were made by independent personnel so that measurement methods did not influence the registered times.

**Figure 5.**
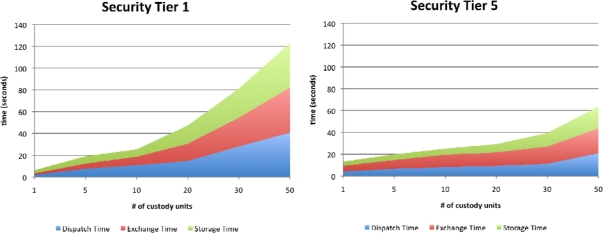
Time results for the Tier 1 and Tier 5 implementations.

Figure 5 shows the time results for the Tier-1 and Tier-5 systems. The vertical axis shows the average dispatch, exchange and storage times obtained in the experiments, while the horizontal axis represents the number of custody units involved in the operations. It can be seen that, for operations involving only a few custody units, preparation and storage times are higher for the Tier-5 system. This is due to the overhead imposed by the custody unit scan control at every operation. However, as the number of custody units increases, simultaneous RFID scan capabilities significantly lower these times, making the repeated scan overhead time negligible compared to the time consumed by the bar code scans. A similar trend can be seen in the exchange and storage times, although the exchange times for Tier-5 systems are slightly higher than storage times due to the need to scan CE employee cards. From these results, we can conclude that the use of RFID scans significantly improves performance (and, therefore, system usability) over the use of bar codes.

Table 3 shows the median failure and error rates measured in the experiments. We can see that the use of RFID greatly contributes to decreased scanning error rates and that performing custody unit scans at every operation effectively reduces the failure rate to zero, thus greatly improving process security.

Finally, Table 4 shows the attack success rates for the different attack experiments. We can see that attack success rates for the experiment where intermediaries are in the same order of magnitude as the failure rates in the previous experiment, since “attack success” in this case is produced by errors in the identification process. However, the success rates are higher than the previous failure rates, since errors are no longer accidental, but induced by the intermediaries. The results of the experiments where the employees consciously tried to ‘cheat’ the system showed that CU scan using bar code does not prevent concealed custody units from leaving the premises, while RFID based scans using GSS effectively mitigate that risk. Regarding the mis-delivery of custody units, we can see that, while for a low number of custody units the attempt to deliver a custody unit to the wrong customer is detected in all cases, as the number of custody unit increases the attack success rate increases in the Tier-1 system. In the Tier-5 system, however, the use of RFID-based PSS-generated receipts makes very difficult to introduce a wrong custody unit in a legitimate delivery, thus effectively mitigating this risk. Only when the number of custody units is very high, a very small attack success rate may be observed.

## 5. Conclusions

Information security and business continuity management are top priorities for today’s medium-to-large scale enterprises. Therefore, many of them rely on thorough backup policies and off-site data storage providers as integrated processes in their business activities to protect their information and to be prepared for emergency recovery if needed. Due to the critical nature of these processes in business continuity and information confidentiality, security is a paramount issue when choosing a solution for off-site data storage.

In this article we have presented SECUR, an RFID-based off-site data storage management system that significantly improves the security of the backup data life cycle. This improvement is due not only to the inherent advantages of RFID over bar code scanners, but also to the deployment of thorough security controls at different points in the processes involved, including state-of–the-art cryptographic mechanisms, the segregation of duties, two- and three-factor authentications and real-time monitoring of custody units. The SECUR system has been successfully implemented and put into production [16], providing added value to customers due to improved security and efficiency. The experimental results presented in this article show the quantifiable part of this improvement as a reduction in the elapsed time and failure rate for custody unit operations.

Though the experiments performed show satisfactory results, there are still future avenues of work and further research needed. We are working on improving scan speeds for encrypted RFID scans so that the gap in usability between security Tiers 6 and 7 is reduced. We are also interested in the design of more cost-effective ISS subsystems so that we can also reduce the significant implementation cost gap between tiers 9 and 10. Finally, electronic vaulting mechanisms [17], along with the growing usage of virtualization and cloud computing techniques in corporate IT infrastructures [18], may have a significant effect on the way data backup needs evolve in the near future. We are interested in leading future research on the SECUR system to address security and usability issues in these scenarios, because that could greatly contribute to improve off-site data storage in the near future.

## Figures and Tables

**Table 1.** Security mechanisms implemented in the different security tiers.

**Security Mechanism**	**Security Tier**										
	0	1	2	3	4	5	6	7	8	9	10
TLS secure connections, username/password, server-side certs., subsystem certs.			✓	✓	✓	✓	✓	✓	✓	✓	✓
Real-time custody unit tracking, browser client-side certificates					✓	✓	✓	✓	✓	✓	✓
Plain RFID identification						✓	✓				
Password protected RFID identification								✓			
Encrypted RFID identification									✓	✓	✓
Vault space division, segregation of duties		✓	✓	✓	✓	✓	✓	✓	✓	✓	✓
PIN-based vault access control		✓	✓	✓	✓						
RFID-based vault access control, RFID-based CU check-in and check-out at vault						✓	✓	✓	✓	✓	✓
RFID-based CU check-in and check-out at the transport vans										✓	✓
Biometric vault access control							✓	✓	✓	✓	✓
Continuous RFID inventory											✓
CU and employee authentication at every operation				✓	✓	✓	✓	✓	✓	✓	✓
Retrieval/delivery receipts		✓									
PSS-generated receipts				✓	✓	✓	✓	✓	✓	✓	✓

**Table 2.** Security risks mitigated in the different security tiers.

**Security Risk**	**Security Tier**										
	0	1	2	3	4	5	6	7	8	9	10
Impersonation of OSSP and CE employees			✓	✓	✓	✓	✓	✓	✓	✓	✓
OSSP and CE employee credential forgery						✓	✓	✓	✓	✓	✓
Custody unit ID tag forgeries						✓	✓	✓	✓	✓	✓
Delivery or retrieval repudiation		✓	✓	✓	✓	✓	✓	✓	✓	✓	✓
Delivery or retrieval to wrong customers			✓	✓	✓	✓	✓	✓	✓	✓	✓
CU exposure to unauthorized customers										✓	✓
Unauthorized CUs leaving the vault						✓	✓	✓	✓	✓	✓
Unauthorized access to the vault		✓	✓	✓	✓	✓	✓	✓	✓	✓	✓
Theft / oversight of credentials for vault access							✓	✓	✓	✓	✓
Custody unit operations by unauthorized personnel				✓	✓	✓	✓	✓	✓	✓	✓
Mistaken/malicious CU operations by authorized pers.					✓	✓	✓	✓	✓	✓	✓
Undue CU manipulations within the vault											✓

**Table 3.** Error and failure rate results.

**# of CU**	**Tier-1**		**Tier-5**	
	**error rate**	**failure rate**	**error rate**	**failure rate**
1	0	0	0	0
5	0	0	0	0
10	0	0	0	0
20	0.01	0.002	0	0
30	0.02	0.005	0.005	0
50	0.05	0.01	0.01	0.0001

**Table 4.** Results for the attack experiments.

	**Intermediary experiment attack success rate (wrong CU leave vault)**		**Conscious attack experiment**			
			**attack success rate (wrong CU leave vault)**		**attack success rate (CU mis-delivered)**	
**# of CU**	**Tier-1**	**Tier-5**	**Tier-1**	**Tier-5**	**Tier-1**	**Tier-5**
1	0	0	1	0	0	0
5	0	0	1	0	0	0
10	0	0	1	0	0.12	0
20	0.004	0	1	0	0.26	0
30	0.007	0	1	0	1	0
50	0.02	0.0002	1	0.0002	1	0.0003

## List of Acronyms

CCSS: Customer Client Subsystem
CE: Customer Enterprise
CEE: CE Employee
CU: Custody Unit
GSS: Gate Subsystem
ISS: Inventory Subsystem
MSS: Management Subsystem
OSSP: Off-site Data Storage Service Provider
OSSPE: OSSP Employee
PIN: Personal Identification Number
PKCS: Public-key Cryptography Standards
PSS: Portable Subsystem

## References

[1] DoD, CSC-STD-001-83 (1983). Trusted Computer System Evaluation Criteria.

[2] Khalfan AM (2004). Information security considerations in IS/IT outsourcing projects: A descriptive case study of two sectors. Int. J. Info. Manage.

[3] Grocholski GT, Noble A (2008). Top Business/Technology Issues Survey Results.

[4] Kermarkar U, Manga V (2006). Business continuity and technology in the retail sector. The Business and Information Technologies (BIT) Project: A Global Study of Business Practice.

[5] Lam W (2002). Ensuring business continuity. IT Prof.

[6] Ward T (2006). Security of backup data. Info. Secur. J. A Glob. Perspective.

[7] Bajgoric N (2009). Continuous Computing Technologies for Enhancing Business Continuity.

[8] Finkenzeller K (2003). RFID Handbook Fundamentals and Applications in Contactless Smart Cards and Identification.

[9] Hodge GM (2000). Best practices for digital archiving: An information life cycle approach. J Electron Publ.

[10] Foley SN (1997). The specification and implementation of commercial security requirements including dynamic segregation of duties.

[11] Rodrigo A, Marsa-Maestre I (2008). Servicio de Custodia Basado en RFID (in Spanish).

[12] Chown P (2002). RFC 3268––Advanced Encryption Standard (AES) Ciphersuites for Transport Layer Security (TLS).

[13] Jonsson J, Kaliski B (2003). RFC 3447––Public-Key Cryptography Standards (PKCS) #1: RSA Cryptography Specifications Version 21.

[14] ISO, ISO/IEC 15693-1to3 Identification cards––Contactless integrated circuit(s) cards––Vicinity cards.

[15] Eloff JH, Eloff M (2005). Information security architecture. Comput. Fraud Secur.

[16] Business continuity management (BSCM) Ltd. corporate web site (in Spanish).

[17] Ganong R (2003). The emergence of e-vaulting. Info. Manage. J.

[18] Mosharaf NM (2010). A survey of network virtualization. Comput. Netw.

